# Ultrasonography as a way of evaluating the diaphragm muscle in patients with chronic obstructive pulmonary disease

**DOI:** 10.1097/MD.0000000000039795

**Published:** 2024-09-20

**Authors:** Bianca Carmo Figueira Silva, Diego Condesso Abreu, Yves Raphael Souza, Manoele Figueiredo, Joseane Felix Macêdo, Thiago Thomaz Mafort, Rogerio Rufino, Claudia Henrique da Costa

**Affiliations:** a Rio de Janeiro State University, Rio de Janeiro, Brazil.

**Keywords:** COPD, diaphragm, diaphragmatic mobility, ultrasonography

## Abstract

The respiratory muscles in chronic obstructive pulmonary disease (COPD) patients have reduced strength and resistance, leading to loss of the functional ability of these muscles. Lung hyperinflation is one of the main alterations, and air trapping is the main factor limiting diaphragmatic mobility (DM) in these patients; however, its correlation with functional parameters, exercise capacity, and indicators of disease severity remains underexplored. This study aimed to assess DM in stable COPD patients and relate the findings with parameters such as the 6-minute walk test distance, forced expiratory volume in 1 second (FEV_1_) %predicted, residual volume (RV) %predicted, and dyspnea. An observational cohort study was conducted to measure DM using ultrasound both at rest (DMrest) and during deep inspiration (DMmax). Forty-nine patients with stable COPD were included in this study. There was a correlation between DMmax and FEV_1_ %predicted (*R* = 0.36; *P* = .012), RV %predicted (r = −0.42; *P* = .01), RV/total lung capacity (r = −0.61; *P* < .001), and distance reached in the 6MWT (*R* = 0.46; *P* = .001). Patients with a modified Medical Research Council score <2 exhibited greater DM than those with a score ≥2 (mean difference = 13.20 ± 4.6 mm; *P* = .0059). Similarly, patients with a Body Mass Index, Airflow Obstruction, Dyspnea and Exercise Capacity index ≤4 showed greater DM (61.95 mm) than those with a Body Mass Index, Airflow Obstruction, Dyspnea and Exercise Capacity index >4 (47.89 mm; mean difference = 14.05 ± 5.3 mm; 95% confidence interval = 25.09–3.01 mm). DM is correlated with bronchial obstruction (FEV_1_), lung hyperinflation (RV and RV/total lung capacity), exercise capacity, and dyspnea in patients with COPD, suggesting its utility as an evaluative tool in this population.

## 1. Introduction

Chronic obstructive pulmonary disease (COPD) is one of the main causes of morbidity and mortality in adults and the elderly worldwide, posing a substantial public health challenge.^[1,2]^ The respiratory muscles in COPD patients have reduced strength and resistance, leading to loss of the functional ability of these muscles.^[3]^ This is due to changes in the rib cage, caused by lung hyperinflation, which alter the area of biomechanical action of the diaphragm muscle, as well as due to systemic factors and structural changes in the respiratory muscles.^[4]^ The diaphragm, as the primary respiratory muscle, undergoes significant changes in contractility and mobility in COPD patients, often exacerbated by lung hyperinflation and air trapping.^[4]^ Lung hyperinflation is one of the main alterations, and air trapping is the main factor limiting diaphragmatic mobility (DM) in these patients.^[4]^ Although the pathophysiological mechanisms involved in the development of dyspnea and poor exercise tolerance in patients with COPD are complex and not well established, it is known that lung hyperinflation plays a central role.^[5]^ However, little is known about the relationship of DM to the degree of bronchial obstruction, lung hyperinflation measured by whole-body plethysmography, or its relationship with exercise capacity. This study aimed to evaluate DM in patients with nonexacerbated COPD and to verify its relationship with functional parameters and exercise capacity.

## 2. Methods

This observational cohort study assessed DM in patients with COPD using ultrasound in a secondary service, at the State University of Rio de Janeiro, Brazil. Patients from the pulmonology outpatient clinic were invited to participate in the study. The diagnosis of COPD was made according to the criteria of the Global Initiative for Chronic Obstructive Lung Disease (GOLD) document.^[2]^ Exclusion criteria included orthopedic limitations affecting the 6-minute walk test (6MWT), neurological disorders, recent (<3 months) cardiovascular events, respiratory infections, other respiratory comorbidities, or the inability to visualize the diaphragm by ultrasound due to anatomical factors.

Exacerbation within the last 2 months was an exclusion criterion; therefore, to avoid creating a classification bias, we decided not to use the clinical criteria. Therefore, we did not use the A, B, and E GOLD classifications but decided to assess severity by forced expiratory volume in 1 second (FEV_1_) (GOLD spirometry classification), degree of dyspnea (modified Medical Research Council [mMRC] scores 0–4), and the Body Mass Index, Airflow Obstruction, Dyspnea and Exercise Capacity (BODE) index. The literature describes the BODE index as an assessment of the severity and mortality risk of patients with COPD.^[6]^ This index not only includes an assessment of the degree of obstruction through FEV_1_ but also uses individual factors such as exercise tolerance (6MWD), body mass index, and dyspnea based on the mMRC score. The scores obtained ranged from 0 to 10, and the higher the patient scores, the worse their condition.^[6]^ The index was calculated as originally proposed,^[6]^ and our patients were divided into 2 groups according to the results of the BODE index (≤4 and >4), where those with higher values were more severe.

The patients underwent the tests on 2 different days. On the first day, a medical questionnaire was filled out, including an assessment of their perception of dyspnea using the mMRC scale.^[7]^ Patients were divided into 2 groups according to the modified mMRC scale. Patients with a score <2 points were considered to have no dyspnea (or to have few symptoms) and those with a score of 2 or more points were considered to have dyspnea. Subsequently, the patient was requested to undergo spirometry to confirm the diagnosis of COPD and finally the 6MWT. Spirometry tests were performed on an HD CPL apparatus (nSpire Health Inc, Longmont, CO) and the American Thoracic Society (ATS) criteria.^[8]^ Forced vital capacity, %predicted, FEV_1_, %predicted, and the FEV_1_/forced vital capacity ratio (%) were determined after 20 minutes of using an inhaled bronchodilator (salbutamol spray, at a dose of 400 µg).^[8]^ The theoretically predicted spirometry values were those described by Knudson et al.^[9]^ The 6MWT was performed according to the guidelines established by ATS in a 30-m corridor,^[10]^ and the 6MWT %predicted was calculated using the equation of Enright and Sherrill.^[11]^

On another day, in the same or the following week, the patient was scheduled to undergo whole-body plethysmography and diaphragmatic ultrasonography. Whole-body plethysmography was performed on an HD CPL apparatus (nSpire Health Inc, Longmont, CO), followed the standardization and interpretation of ATS,^[12]^ and the Neder equations were adopted.^[13]^ In 2020, due to the COVID-19 pandemic, we stopped performing whole-body plethysmography on patients, according to the guidance received from the Brazilian Society of Pulmonology. Thus, the patients recruited during or after 2020 did not undergo this test.

Ultrasound examination was performed by a single specialized and trained professional using a TOSHIBA device (model SSA-370ª Power Vision 6.000). To assess DM, the patient was placed in the supine position, and a 3.5-MHz convex transducer was used, which allowed the observation of deeper structures. This transducer was positioned in the right subcostal region of the midclavicular line, with an incidence angle perpendicular to the craniocaudal axis and diaphragm. This was then identified through the hepatic acoustic window, and its mobility was assessed by measuring, in millimeters (through M mode), its craniocaudal displacement during breathing at rest (tidal volume). This was labeled as DMrest. Then, the measurement was repeated considering the incursion performed from the end of quiet exhalation (functional residual capacity level) to the end of deep inspiration (total lung capacity [TLC] level). Three serial measurements were performed, and the highest value was recorded as the DMmax. The transducer was positioned to observe the entire movement of diaphragmatic excursion. This technique has been described previously and is widely used.^[14,15]^ All examinations were performed on the right side to take advantage of the acoustic window of the liver, which facilitates diaphragmatic visualization.

Statistical analyses were performed using Prism 9.2. The normality of the sample data was confirmed using the Kolmogorov–Smirnov test. Continuous data are presented as the mean and standard deviation. The relationships between continuous variables were determined using Pearson correlation coefficients. Differences between groups were determined using an unpaired *t* test with Welch correction. Results with a *P* value of <0.05 were considered statistically significant.

### 2.1. Sample size calculation

The sample size calculation was based on the correlation between DM and the functional parameters as previously reported.^[16]^ Considering an α-error of 5% and β of 20% with a correlation coefficient of 0.5, the number of patients required would be 29. If the correlation coefficient was 0.4, the required number would be 47. Therefore, considering the possibility of dropout, we planned to recruit 49 patients.

This study was approved by the Local Ethics Committee (No. 82764817.8.0000.5259). All individuals signed a free will and informed consent form before participating in the study procedures in compliance with the Declaration of Helsinki.

## 3. Results

**Figure 1.**
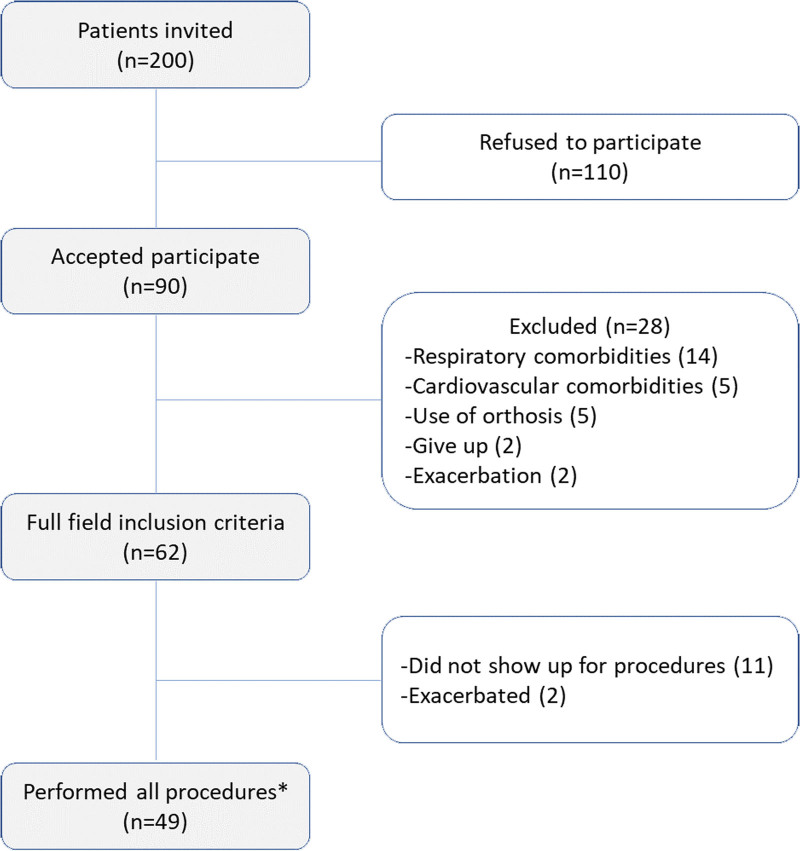
Patient recruitment flowchart. In 2020, owing to the COVID-19 pandemic, we stopped performing whole-body plethysmography on patients according to the guidance received from the Brazilian Society of Pulmonology. Thus, the data presented on these measures account for the first 36 patients recruited before the pandemic who had already performed all study procedures. * Procedures performed: assessment of dyspnea using the modified Medical Research Council scale, diaphragmatic mobility by ultrasound, spirometry, and the 6-minute walk test.

Patient recruitment was performed as shown in the flowchart (Fig. 1). The characteristics of the 49 patients included in the study are presented in Table 1. We then verified that 31 patients (63%) continued to complain of dyspnea despite pharmacological treatment and that 27 (55%) were classified as spirometric GOLD 3 or 4, indicating disease severity in the study group.

**Table 1:** Patient data (n = 49).

Outcomes	Values
Gender (M/F)	27 (55%)/22 (45%)
Age (yr)	69.27 ± 7.55
Weight (kg)	71.65 ± 16.32
BMI (kg/m^2^)	27.40 ± 5.80
mMRC score (n; %)	
0	4; 8
1	14; 29
2	15; 31
3	5; 10
4	11; 22
FEV_1_ (%predicted)	53.86 ± 21.09
FVC (%predicted)	81.51 ± 23.67
FEV_1_/FVC (%)	49.71 ± 12.30
6MWT	
Distance (m) %Predicted	384 ± 96.91 79.05 ± 23.36
Classification GOLD (n; %)	
1	7; 14
2	15; 31
3	21; 43
4	6; 12
DMrest (mm)	31.10 ± 11.87
DMmax (mm)	59.41 ± 17.91

Values are expressed as mean ± standard deviation or n (%).

6MWT = 6-minute walk test, BMI = body mass index, DMmax = diaphragmatic mobility measured from functional residual capacity to the end of a deep inspiration, DMrest = diaphragmatic mobility at rest, F = female, FEV_1_ = forced expiratory volume in 1 second, FVC = forced vital capacity, GOLD = Global Initiative for Chronic Obstructive Lung Disease, M = male, mMRC = modified Medical Research Council.

Table 1 shows the means and standard deviations referring to measures of DMrest and DMmax. When comparing the DM measurements obtained with the patient at rest with those obtained during deep inspiration, we found a positive correlation (*R* = 0.63; 95% confidence interval [CI] = 0.43–0.78; *P* < .0001).

**Figure 2.**
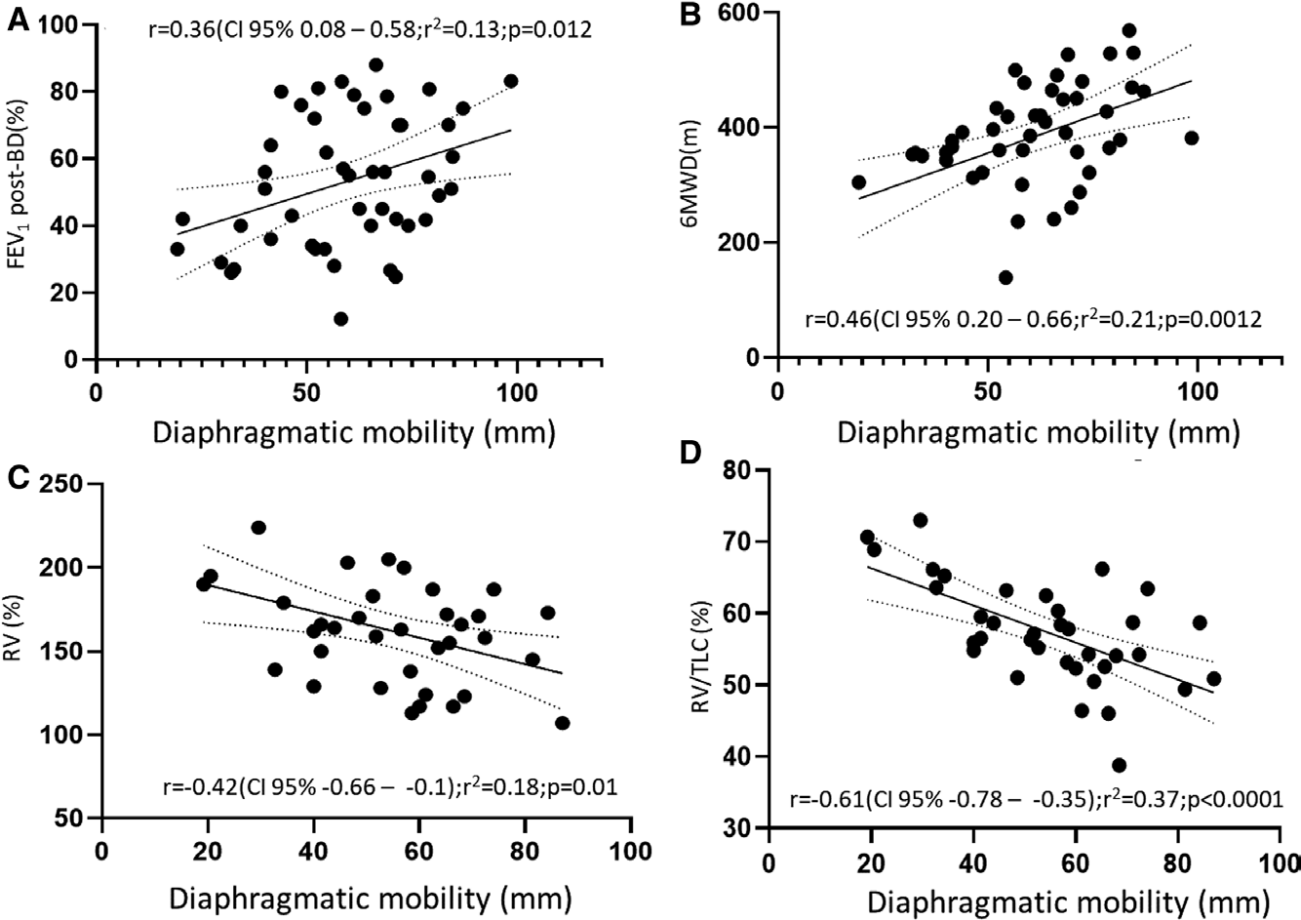
Correlation of DM with functional variables. (A–D) Correlation of DM with exercise capacity, respiratory obstruction, and hyperinflation. 6MWD = 6-minute walk distance, BD = bronchodilation, CI = confidence interval, DM = diaphragmatic mobility, FEV_1_ = forced expiratory volume in the first second, RV = residual volume, TLC = total lung capacity.

Table 2 shows the correlations observed between the main variables studied and DM evaluated at maximum inspiration and at rest. We observed a weak but statistically significant correlation (*R* = 0.36; 95% CI = 0.08–0.58; *P* = .012) between FEV_1_ and DMmax. This graph is shown in Figure 2A.

**Table 2:** Correlation between diaphragmatic mobility and functional test results.

Outcome	r	95% CI	*P* value
Correlation with DMmax			
FEV_1_ (%predicted, post BD)	0.36	0.08 to 0.58	**0.012**
FVC (%predicted, post BD)	0.17	−0.11 to 0.40	0.24 (ns)
RV (%predicted)	−0.42	−0.66 to 0.0	**0.01**
RV/TLC (%)	−0.61	−0.78 to − 0.35	**<0.001**
6MWD (m)	0.46	0.20 to 0.66	**0.001**
Correlation with DMrest			
FEV_1_ (%predicted, post BD)	0.08	−0.20 to 0.35	0.56 (ns)
FVC (%predicted, post BD)	−0.07	−0.35 to 0.21	0.60 (ns)
RV (L)	−0.06	−0.27 to −0.37	0.74 (ns)
RV/TLC (%)	−0.15	−0.45 to 0.18	0.38 (ns)
6MWD (m)	0.30	0.02 to 0.50	**0.03**

Values are expressed as correlation (r) and 95% CI. *P* values < .05 were considered statistically significant.

6MWD = 6-minute walk distance, BD = bronchodilation, CI = confidence interval, DMmax = diaphragmatic mobility measured from the end of quiet to the end of deep inspiration, DMrest = diaphragmatic mobility at rest, FEV_1_ = forced expiratory volume in the first second, FVC = forced vital capacity, ns = not significant, RV = residual volume, TLC = total lung capacity.

We observed a moderate and statistically significant relationship between the 6MWD achieved and DM, both when measured at rest (DMrest), as shown in Table 2, and at maximal inspiration (Table 2; Fig. 2B). Thus, the patients with the best performance in the field test (6MWT) had the highest DM values.

We found a weak but statistically significant correlation between DMmax and residual volume (RV) %predicted (r = −0.42; 95% CI = −0.66 to 0.0; *P* = .01) and a moderate and statistically significant correlation between DMmax and RV/TLC %predicted (r = −0.61; 95% CI = −0.78 to −0.35; *P* < .001). This means that patients with greater hyperinflation had a lower DM. Figure 2C shows the correlation of DMmax with the RV %predicted values and Figure 2D, with the RV/TLC %predicted values.

**Figure 3.**
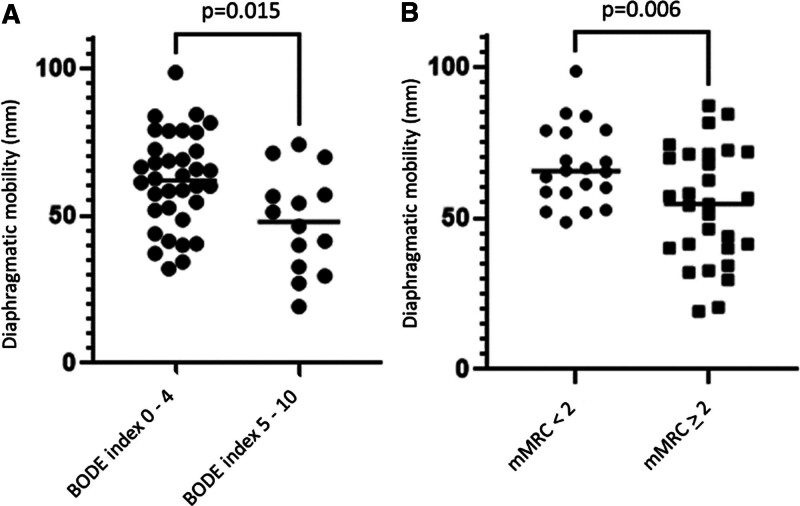
DM according to the BODE index and dyspnea. (A and B) Relationship of DM with dyspnea. BODE = Body Mass Index, Airflow Obstruction, Dyspnea and Exercise Capacity, DM = diaphragmatic mobility, mMRC, modified Medical Research Council.

Most symptomatic patients (mMRC score ≥ 2) had lower DMmax values (54.02 mm) than those without dyspnea (67.23 mm). The difference in means was 13.20 ± 4.6 (95% CI = 22.41–3.99) mm, and this difference was statistically significant (*P* = .0059), as illustrated in Figure 3.

We found a statistically significant difference (*P* = .015) between the mean DMmax of patients with a BODE index ≤4 (61.95 mm) when compared with the results obtained from patients with a BODE index >4 (47.89 mm). The difference between the means was 14.05 ± 5.3 (95% CI = 25.09–3.01) mm. The graph is shown in Figure 3.

In a multivariate analysis, 6MWT (OR = 0.03512; 95% CI = 0.01–0.15; *P* = 0.02), RV (%predicted; OR = 0.43; 95% CI = 0.02–0.84; *P* = .04), and RV/TLC (OR = −2.44; 95% CI = −4.49 to −0.39; *P* = .02) were associated to DMmax.

## 4. Discussion

This study investigated the relationship between DM and key variables assessing airway obstruction, particularly FEV_1_, lung hyperinflation (RV and RV/TLC ratio), exercise capacity, and dyspnea severity in patients with stable COPD. We found significant correlations between these parameters and DMmax, and the multivariate analysis confirmed the association of DM with 6MWT and variables related to hyperinflation. Our results suggest that patients with a lower DMmax tend to have lower FEV_1_, indicating more severe obstruction and increased hyperinflation. Additionally, they exhibited reduced exercise capacity and higher levels of dyspnea.

Previous studies have explored the association between DM and pulmonary function^[16–18]^; however, information regarding the correlation between diaphragmatic excursion and lung hyperinflation in patients with stable COPD is limited. For instance, Chen et al^[16]^ suggested that lung ultrasound scores may better diagnose severe lung hyperinflation compared to DM at maximal deep inspiration. Schulz et al,^[17]^ however, recently published findings supporting a strong correlation between DM and RV.

Rocha et al^[18]^ used chest radiography to evaluate DM in COPD patients and found moderate correlations with FEV_1_ and strong correlations with inspiratory capacity. Despite methodological differences, their results align closely with ours. A meta-analysis involving ultrasound studies in patients with COPD reported lower DM in severe COPD than in mild-to-moderate cases, although this difference was not statistically significant.^[19]^

Another recent study examining 55 supine COPD patients using ultrasound observed reduced diaphragmatic excursion during exacerbations (from 40.4 to 30.8 mm), suggesting that factors beyond bronchial obstruction influence diaphragmatic function.^[20]^ While all our patients were stable during the evaluation, those with poorer performance on 6MWT and higher BODE scores demonstrated lower DM.

Our study found correlations between the BODE index and 6MWT with DMmax, suggesting that sonographically measured DM correlates with different parameters related to COPD severity. Other studies performed in COPD patients with lung hyperinflation reported a correlation between DM and exercise tolerance.^[21]^ Using the same methodology, our study confirmed the relationship between exercise capacity and DM even in stable COPD patients without significant hyperinflation. A recent study highlighted the correlation between DM and the ventilation parameters assessed during cardiopulmonary exercise testing.^[22]^ This study suggests that reduced DM might contribute to decreased exercise capacity and heightened dyspnea in patients with COPD, potentially due to dynamic lung hyperinflation. These findings underscore the impact of diaphragmatic function on exercise tolerance and respiratory symptoms in individuals with COPD.^[22]^

Shen et al^[23]^ evaluated DM in 63 patients diagnosed with COPD and obstructive sleep apnea overlap syndrome who actively participated in pulmonary rehabilitation. The authors reported that the changes in 6MWD were positively associated with baseline DM during deep breathing and negatively correlated with dyspnea, concluding that diaphragm excursion could predict exercise tolerance in these patients.^[23]^

Interestingly, a study by Rocha et al^[18]^ using radiography in forced inhalation and exhalation correlated the findings of DM measurements with dyspnea assessed using the mMRC scale. Likewise, our study, which evaluated DM through ultrasound, also achieved the same results. Symptomatic patients generally had a lower DM (*P* = .0059). Our study included 18 patients with mMRC scores 0 and 1 and 31 patients complaining of dyspnea (mMRC score > 2), which made it possible to evaluate the association of this symptom with DM. It is known that the cause of dyspnea is multifactorial, but it is likely that lung hyperinflation and muscle weakness found in more symptomatic patients are responsible for the reduction in MD.

Correlations between DM and functional parameters were negative when measured at rest (DMrest), potentially because of the smaller diaphragmatic muscle displacement during restful breathing. Alternatively, hyperinflation may be more evident during maximal inspiration (DMmax), affecting ventilation less at the tidal volume and more at maximal excursion. Lowering the diaphragm in these patients would allow ventilation at the tidal volume level to be less impacted, whereas a wider excursion, measured in maximal inspiration, would be more compromised.

With recent advances in ultrasound technology, studies on diaphragmatic muscle have increased, with some examining its relationship with pulmonary function in non-COPD individuals.^[24]^ However, understanding DM in relation to lung function and COPD severity remains incomplete. We chose ultrasound for its practicality in assessing diaphragmatic muscles, highlighting its portability, cost-effectiveness, and lack of contraindications even in severe COPD.

Due to the widespread use of ultrasound, the data obtained here suggest that the MD measurement may be another reliable variable to be used in the monitoring of patients with stable COPD. However, the limitations of our study include its single-center nature with a sample of patients with relatively severe illness, as well as restricted whole-body plethysmography due to the COVID-19 pandemic. The lack of long-term follow-up limits the prognostic assessment despite promising results, emphasizing the need for further studies. Our study used 1 professional trained in thoracic ultrasound examination to maintain the consistency of the assessment. Although this professional is experienced in the technique, there was no checking of the measurements carried out by a second person.

## 5. Conclusion

Diaphragmatic excursion measured during deep inspiration correlates with airway obstruction, pulmonary hyperinflation, dyspnea severity, and functional capacity in patients with COPD. This measurement could serve as a biomarker for assessing the disease severity in clinical practice.

## Author contributions

**Investigation:** Bianca Carmo Figueira Silva, Diego Condesso Abreu, Joseane Felix Macêdo.

**Methodology:** Bianca Carmo Figueira Silva, Manoele Figueiredo, Rogerio Rufino.

**Writing – original draft:** Bianca Carmo Figueira Silva.

**Data curation:** Diego Condesso Abreu, Yves Raphael Souza.

**Visualization:** Yves Raphael Souza.

**Conceptualization:** Thiago Thomaz Mafort, Rogerio Rufino, Claudia Henrique da Costa.

**Formal analysis:** Thiago Thomaz Mafort.

**Writing – review & editing:** Thiago Thomaz Mafort, Claudia Henrique da Costa.

**Funding acquisition:** Claudia Henrique da Costa.

**Supervision:** Claudia Henrique da Costa.
